# μ-Bis(diphenyl­arsino)methane-1:2κ^2^ *As*:*As*-nona­carbonyl-1κ^3^ *C*,2κ^3^ *C*,3κ^3^ *C*-[(2-methoxy­phen­yl)diphenyl­phosphino-3κ*P*]-*triangulo*-triruthenium(0)

**DOI:** 10.1107/S1600536809047783

**Published:** 2009-11-21

**Authors:** Omar bin Shawkataly, Imthyaz Ahmed Khan, Chin Sing Yeap, Hoong-Kun Fun

**Affiliations:** a Chemical Sciences Programme, School of Distance Education, Universiti Sains Malaysia, 11800 USM, Penang, Malaysia; b X-ray Crystallography Unit, School of Physics, Universiti Sains Malaysia, 11800 USM, Penang, Malaysia

## Abstract

In the title *triangulo*-triruthenium compound, [Ru_3_(C_25_H_22_As_2_)(C_19_H_17_OP)(CO)_9_], the bis­(diphenyl­arsino)methane ligand bridges a Ru—Ru bond and the monodentate phosphine ligand bonds to the third Ru atom. Both the phosphine and arsine ligands are equatorial with respect to the Ru_3_ triangle. Additionally, each Ru atom carries one equatorial and two axial terminal carbonyl ligands. The three phosphine substituted phenyl rings make dihedral angles of 74.34 (12), 68.34 (12) and 85.45 (11)° with each other. The dihedral angles between the two phenyl rings are 87.56 (11) and 60.56 (11)° for the two diphenyl­arsino groups. In the crystal packing, the mol­ecules are linked together into chains *via* inter­molecular C—H⋯O hydrogen bonds down the *a* axis. Weak inter­molecular C—H⋯π inter­actions further stabilize the crystal structure.

## Related literature

For general background to *triangulo*-triruthenium derivatives, see: Bruce *et al.* (1985 ▶, 1988*a* ▶,*b* ▶); Shawkataly *et al.* (1998 ▶, 2004 ▶, 2009*a* ▶,*b* ▶). For related structures, see: Shawkataly *et al.* (2009*a* ▶,*b* ▶). For the synthesis of bis­(diphenylarsino)methane, see: Bruce *et al.* (1983 ▶). For the stability of the temperature controller used for the data collection, see: Cosier & Glazer (1986 ▶).
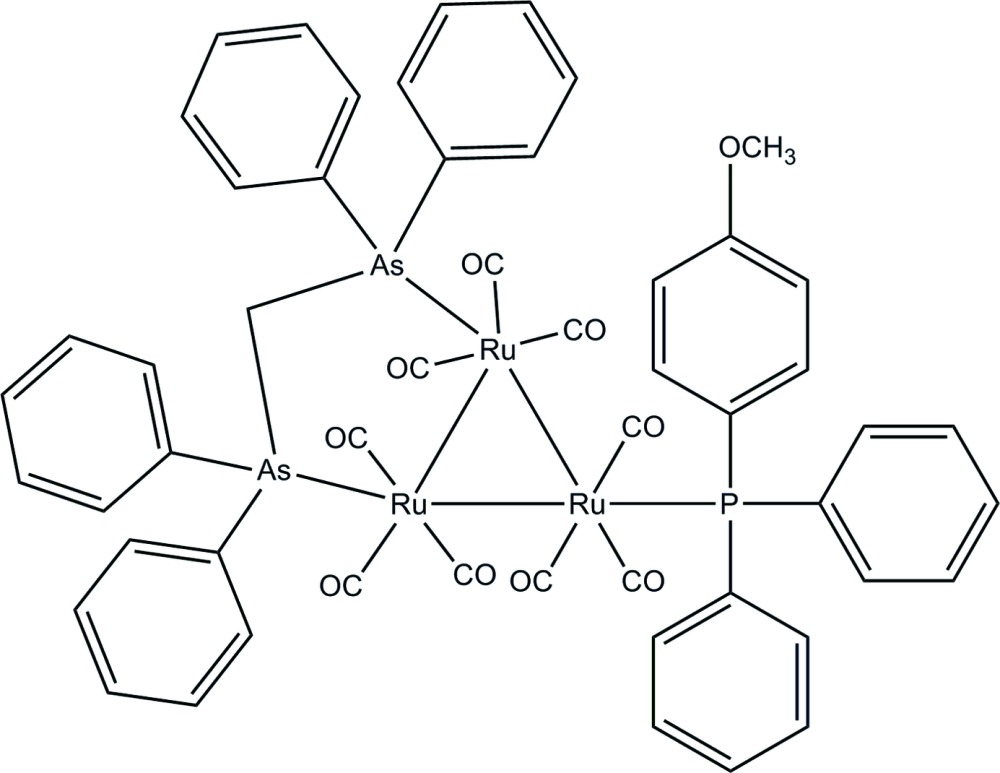


## Experimental

### 

#### Crystal data

[Ru_3_(C_25_H_22_As_2_)(C_19_H_17_OP)(CO)_9_]
*M* *_r_* = 1319.86
Triclinic, 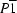

*a* = 10.4414 (1) Å
*b* = 11.6541 (1) Å
*c* = 21.7581 (3) Å
α = 84.220 (1)°
β = 84.910 (1)°
γ = 69.324 (1)°
*V* = 2460.49 (5) Å^3^
*Z* = 2
Mo *K*α radiation
μ = 2.34 mm^−1^
*T* = 100 K
0.22 × 0.15 × 0.08 mm

#### Data collection

Bruker SMART APEXII CCD area-detector diffractometer
Absorption correction: multi-scan (**SADABS**; Bruker, 2005 ▶) *T*_min_ = 0.624, *T*_max_ = 0.830
66801 measured reflections
14256 independent reflections
11583 reflections with *I* > 2σ(*I*)
*R*_int_ = 0.042

#### Refinement

*R*[*F* ^2^ > 2σ(*F* ^2^)] = 0.027
*wR*(*F* ^2^) = 0.059
*S* = 1.02
14256 reflections
623 parameters
H-atom parameters constrained
Δρ_max_ = 0.67 e Å^−3^
Δρ_min_ = −0.80 e Å^−3^

### 

Data collection: *APEX2* (Bruker, 2005 ▶); cell refinement: *SAINT* (Bruker, 2005 ▶); data reduction: *SAINT*; program(s) used to solve structure: *SHELXTL* (Sheldrick, 2008 ▶); program(s) used to refine structure: *SHELXTL*; molecular graphics: *SHELXTL*; software used to prepare material for publication: *SHELXTL* and *PLATON* (Spek, 2009 ▶).

## Supplementary Material

Crystal structure: contains datablocks global, I. DOI: 10.1107/S1600536809047783/sj2676sup1.cif

Structure factors: contains datablocks I. DOI: 10.1107/S1600536809047783/sj2676Isup2.hkl

Additional supplementary materials:  crystallographic information; 3D view; checkCIF report

## Figures and Tables

**Table 1:** Hydrogen-bond geometry (Å, °)

*D*—H⋯*A*	*D*—H	H⋯*A*	*D*⋯*A*	*D*—H⋯*A*
C16—H16*A*⋯O4^i^	0.93	2.52	3.244 (3)	135
C29—H29*A*⋯O5^ii^	0.93	2.56	3.286 (3)	135
C5—H5*A*⋯*Cg*1^iii^	0.93	2.84	3.668 (2)	149
C18—H18*A*⋯*Cg*2^iv^	0.93	2.95	3.701 (2)	139
C22—H22*A*⋯*Cg*2^v^	0.93	2.95	3.787 (2)	151
C24—H24*A*⋯*Cg*3^iv^	0.93	2.81	3.499 (2)	132

Symmetry codes: (i) 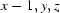; (ii) 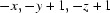; (iii) 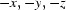; (iv) 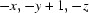; (v) 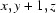. *Cg*1, *Cg*2 and *Cg*3 are the centroids of the C7–C12, C1–C6 and C14–C19 rings, respectively.

**Fig. 1.**
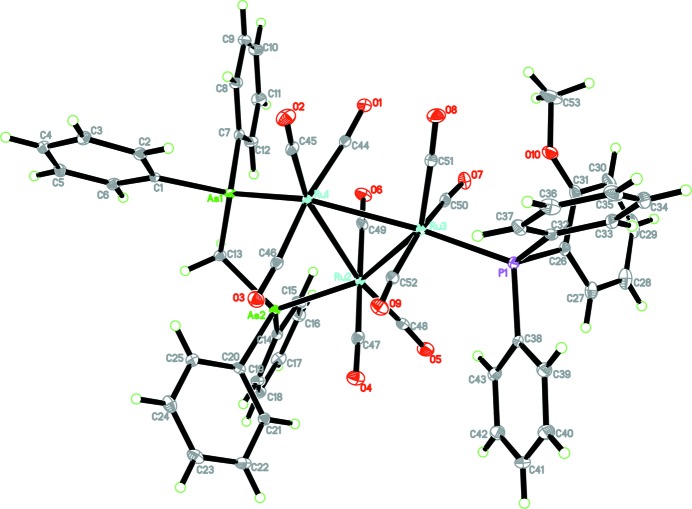
The molecular structure of the title compound with 30% probability ellipsoids for non-H atoms.

**Fig. 2.**
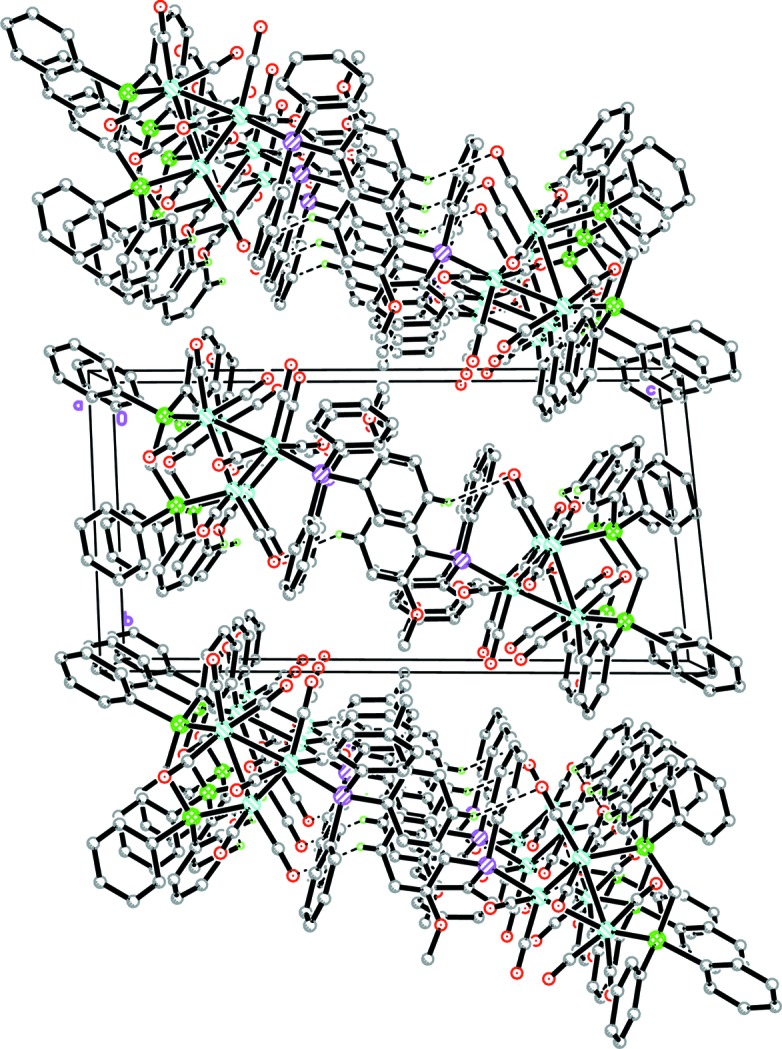
The crystal packing of the title compound, viewed down the a axis, showing the molecules linked down a axis. Hydrogen atoms that not involved in the hydrogen-bonding (dashed lines) have been omitted for clarity.

## supplementary crystallographic information

### Comment

Tri-angulotriruthenium clusters are known for their interesting structural variations and related catalytic activity. A large number of substituted derivatives, Ru_3_(CO)_12-n_*L*_n_ (*L* = group 15 ligand) have been reported (Bruce *et al.*, 1985 1988a,b;). As part of our study on the substitution of transition metal-carbonyl clusters with mixed-ligand complexes, we have published several structures of tri-angulotriruthenium-carbonyl clusters containing mixed P/As and P/Sb ligands (Shawkataly *et al.*, 1998, 2004, 2009a,b). Herein we report the synthesis and structure of Ru_3_(C_19_H_17_OP)(C_25_H_22_As_2_)(CO)_9_.

The bond lengths and angles of title compound (Fig. 1) are comparable to those in related structures (Shawkataly *et al.*, 2009a,b). The bis(diphenylarsino)methane ligand bridges the Ru1—Ru2 bond and the monodentate phosphine ligand bonds to the Ru3 atom. Both the phosphine and arsine ligands are equatorial with respect to the Ru_3_ triangle. Additionally, each Ru atom carries one equatorial and two axial terminal carbonyl ligands. The three phosphine substituted phenyl rings make dihedral angles (C26–C31/C32–C37, C26–C31/C38–C43 and C32–C37/C38–C43) of 74.34 (12), 68.34 (12) and 85.45 (11)° with each other respectively. The dihedral angles between the two phenyl rings (C1–C6/C7–C12 and C14–C19/C20–C25) are 87.56 (11) and 60.56 (11)° for the two diphenylarsino groups respectively.

In the crystal packing (Fig. 2), the molecules are linked together into chains *via* intermolecular C16—H16A···O4 and C29—H29A···O5 hydrogen bonds down *a* axis. Weak intermolecular C—H···π interaction further stabilize the crystal structure (Table 1).

### Experimental

The reactions were conducted under an atmosphere of nitrogen using standard Schlenk techniques and hexane-dried over sodium metal. (2-Methoxyphenyl)diphenylphosphine (Strem Chemicals) was used as received and bis(diphenylarsino)methane (Bruce *et al.*, 1983) was prepared by the reported procedure. The title compound was obtained by refluxing equimolar quantities of Ru_3_(CO)_10_(µ-Ph_2_AsCH_2_AsPh_2_) (105.5 mg, 0.1 mmol) and (2-methoxyphenyl)diphenylphosphine (29.23 mg, 0.1 mmol) in hexane under a nitrogen atmosphere. Crystals suitable for X-ray diffraction were grown by slow solvent / solvent diffusion of CH_3_OH into CHCl_3_.

### Refinement

All hydrogen atoms were positioned geometrically and refined using a riding model with C—H = 0.93–0.97 Å and *U*_iso_(H) = 1.2 or 1.5 *U*_eq_(C). A rotating group model was used for the methyl groups.

### Crystal data

**Table:**

[Ru_3_(C_25_H_22_As_2_)(C_19_H_17_OP)(CO)_9_]	*Z* = 2
*M**_r_* = 1319.86	*F*(000) = 1300
Triclinic, *P*1	*D*_x_ = 1.782 Mg m^−^^3^
Hall symbol: -P 1	Mo *K*α radiation, λ = 0.71073 Å
*a* = 10.4414 (1) Å	Cell parameters from 9969 reflections
*b* = 11.6541 (1) Å	θ = 2.3–32.5°
*c* = 21.7581 (3) Å	µ = 2.34 mm^−^^1^
α = 84.220 (1)°	*T* = 100 K
β = 84.910 (1)°	Block, purple
γ = 69.324 (1)°	0.22 × 0.15 × 0.08 mm
*V* = 2460.49 (5) Å^3^	

### Data collection

**Table:**

Bruker SMART APEXII CCD area-detector diffractometer	14256 independent reflections
Radiation source: fine-focus sealed tube	11583 reflections with *I* > 2σ(*I*)
graphite	*R*_int_ = 0.042
φ and ω scans	θ_max_ = 30.0°, θ_min_ = 1.9°
Absorption correction: multi-scan (*SADABS*; Bruker, 2005)	*h* = −14→14
*T*_min_ = 0.624, *T*_max_ = 0.830	*k* = −16→16
66801 measured reflections	*l* = −30→30

### Refinement

**Table:**

Refinement on *F*^2^	Primary atom site location: structure-invariant direct methods
Least-squares matrix: full	Secondary atom site location: difference Fourier map
*R*[*F*^2^ > 2σ(*F*^2^)] = 0.027	Hydrogen site location: inferred from neighbouring sites
*wR*(*F*^2^) = 0.059	H-atom parameters constrained
*S* = 1.02	*w* = 1/[σ^2^(*F*_o_^2^) + (0.0249*P*)^2^ + 1.1204*P*] where *P* = (*F*_o_^2^ + 2*F*_c_^2^)/3
14256 reflections	(Δ/σ)_max_ = 0.001
623 parameters	Δρ_max_ = 0.67 e Å^−^^3^
0 restraints	Δρ_min_ = −0.80 e Å^−^^3^

### Special details

**Table:**

Experimental. The crystal was placed in the cold stream of an Oxford Cyrosystems Cobra open-flow nitrogen cryostat (Cosier & Glazer, 1986) operating at 100.0 (1) K.
Geometry. All e.s.d.'s (except the e.s.d. in the dihedral angle between two l.s. planes) are estimated using the full covariance matrix. The cell e.s.d.'s are taken into account individually in the estimation of e.s.d.'s in distances, angles and torsion angles; correlations between e.s.d.'s in cell parameters are only used when they are defined by crystal symmetry. An approximate (isotropic) treatment of cell e.s.d.'s is used for estimating e.s.d.'s involving l.s. planes.
Refinement. Refinement of *F*^2^ against ALL reflections. The weighted *R*-factor *wR* and goodness of fit *S* are based on *F*^2^, conventional *R*-factors *R* are based on *F*, with *F* set to zero for negative *F*^2^. The threshold expression of *F*^2^ > σ(*F*^2^) is used only for calculating *R*-factors(gt) *etc*. and is not relevant to the choice of reflections for refinement. *R*-factors based on *F*^2^ are statistically about twice as large as those based on *F*, and *R*- factors based on ALL data will be even larger.

### Fractional atomic coordinates and isotropic or equivalent isotropic displacement parameters (Å^2^)

**Table:**

	*x*	*y*	*z*	*U*_iso_*/*U*_eq_	
Ru1	0.304381 (17)	0.169488 (15)	0.205414 (8)	0.01208 (4)	
Ru2	0.134145 (17)	0.409411 (15)	0.239146 (8)	0.01210 (4)	
Ru3	0.366826 (17)	0.266015 (15)	0.309439 (8)	0.01236 (4)	
As1	0.14602 (2)	0.160785 (19)	0.131363 (10)	0.01190 (5)	
As2	0.03462 (2)	0.457750 (19)	0.139265 (10)	0.01170 (5)	
P1	0.42521 (6)	0.35723 (5)	0.38908 (3)	0.01382 (11)	
O1	0.17427 (18)	0.03586 (15)	0.30752 (7)	0.0228 (4)	
O2	0.55893 (18)	−0.06037 (15)	0.20185 (8)	0.0273 (4)	
O3	0.45546 (17)	0.30219 (15)	0.11329 (8)	0.0234 (4)	
O4	0.33593 (17)	0.53968 (15)	0.19120 (8)	0.0236 (4)	
O5	−0.0492 (2)	0.64365 (15)	0.29555 (8)	0.0298 (4)	
O6	−0.07509 (17)	0.28542 (16)	0.28668 (8)	0.0242 (4)	
O7	0.11632 (17)	0.25538 (16)	0.39212 (7)	0.0223 (4)	
O8	0.53996 (19)	0.00676 (15)	0.35308 (8)	0.0291 (4)	
O9	0.59248 (17)	0.33052 (15)	0.22840 (8)	0.0228 (4)	
O10	0.35430 (18)	0.17638 (16)	0.47263 (8)	0.0246 (4)	
C1	0.2071 (2)	0.07700 (19)	0.05505 (10)	0.0134 (4)	
C2	0.3397 (2)	−0.0067 (2)	0.04739 (10)	0.0164 (4)	
H2A	0.4021	−0.0186	0.0775	0.020*	
C3	0.3793 (2)	−0.0725 (2)	−0.00503 (10)	0.0182 (4)	
H3A	0.4676	−0.1293	−0.0097	0.022*	
C4	0.2873 (2)	−0.0534 (2)	−0.05045 (10)	0.0182 (5)	
H4A	0.3145	−0.0966	−0.0858	0.022*	
C5	0.1552 (2)	0.0296 (2)	−0.04332 (10)	0.0180 (4)	
H5A	0.0938	0.0426	−0.0739	0.022*	
C6	0.1146 (2)	0.0933 (2)	0.00947 (10)	0.0167 (4)	
H6A	0.0250	0.1473	0.0147	0.020*	
C7	0.0285 (2)	0.0696 (2)	0.16641 (10)	0.0149 (4)	
C8	0.0956 (2)	−0.0566 (2)	0.18016 (10)	0.0175 (4)	
H8A	0.1891	−0.0918	0.1703	0.021*	
C9	0.0238 (2)	−0.1294 (2)	0.20841 (10)	0.0201 (5)	
H9A	0.0692	−0.2130	0.2175	0.024*	
C10	−0.1162 (3)	−0.0774 (2)	0.22309 (11)	0.0224 (5)	
H10A	−0.1649	−0.1261	0.2417	0.027*	
C11	−0.1828 (3)	0.0478 (2)	0.20972 (11)	0.0225 (5)	
H11A	−0.2763	0.0828	0.2195	0.027*	
C12	−0.1106 (2)	0.1215 (2)	0.18172 (10)	0.0183 (4)	
H12A	−0.1558	0.2053	0.1733	0.022*	
C13	0.0169 (2)	0.32006 (19)	0.09980 (10)	0.0145 (4)	
H13A	0.0341	0.3310	0.0554	0.017*	
H13B	−0.0761	0.3202	0.1074	0.017*	
C14	−0.1539 (2)	0.56912 (19)	0.13745 (10)	0.0142 (4)	
C15	−0.2506 (2)	0.5387 (2)	0.17774 (11)	0.0192 (5)	
H15A	−0.2222	0.4697	0.2055	0.023*	
C16	−0.3882 (2)	0.6101 (2)	0.17687 (11)	0.0212 (5)	
H16A	−0.4521	0.5889	0.2037	0.025*	
C17	−0.4304 (2)	0.7133 (2)	0.13582 (12)	0.0223 (5)	
H17A	−0.5231	0.7604	0.1343	0.027*	
C18	−0.3347 (2)	0.7466 (2)	0.09695 (11)	0.0222 (5)	
H18A	−0.3632	0.8174	0.0703	0.027*	
C19	−0.1965 (2)	0.6748 (2)	0.09750 (10)	0.0172 (4)	
H19A	−0.1327	0.6974	0.0713	0.021*	
C20	0.1329 (2)	0.5313 (2)	0.07702 (10)	0.0144 (4)	
C21	0.1333 (2)	0.6484 (2)	0.08547 (10)	0.0165 (4)	
H21A	0.0794	0.6922	0.1176	0.020*	
C22	0.2144 (2)	0.6995 (2)	0.04593 (11)	0.0205 (5)	
H22A	0.2138	0.7778	0.0514	0.025*	
C23	0.2957 (2)	0.6342 (2)	−0.00136 (11)	0.0230 (5)	
H23A	0.3502	0.6685	−0.0276	0.028*	
C24	0.2965 (2)	0.5178 (2)	−0.00993 (11)	0.0226 (5)	
H24A	0.3513	0.4740	−0.0419	0.027*	
C25	0.2148 (2)	0.4663 (2)	0.02946 (10)	0.0180 (4)	
H25A	0.2154	0.3880	0.0237	0.022*	
C26	0.3085 (2)	0.3882 (2)	0.45772 (10)	0.0171 (4)	
C27	0.2424 (2)	0.5067 (2)	0.47683 (11)	0.0225 (5)	
H27A	0.2619	0.5722	0.4553	0.027*	
C28	0.1483 (3)	0.5289 (3)	0.52739 (12)	0.0303 (6)	
H28A	0.1052	0.6084	0.5396	0.036*	
C29	0.1194 (3)	0.4319 (3)	0.55912 (12)	0.0336 (7)	
H29A	0.0549	0.4467	0.5924	0.040*	
C30	0.1848 (3)	0.3124 (3)	0.54234 (11)	0.0307 (6)	
H30A	0.1649	0.2476	0.5645	0.037*	
C31	0.2803 (3)	0.2903 (2)	0.49224 (11)	0.0225 (5)	
C32	0.5915 (2)	0.2698 (2)	0.42165 (10)	0.0165 (4)	
C33	0.6086 (3)	0.2668 (2)	0.48475 (11)	0.0215 (5)	
H33A	0.5345	0.3067	0.5111	0.026*	
C34	0.7365 (3)	0.2039 (2)	0.50829 (12)	0.0262 (5)	
H34A	0.7470	0.2006	0.5505	0.031*	
C35	0.8479 (3)	0.1465 (2)	0.46937 (12)	0.0263 (5)	
H35A	0.9335	0.1062	0.4853	0.032*	
C36	0.8325 (2)	0.1487 (2)	0.40697 (12)	0.0251 (5)	
H36A	0.9074	0.1097	0.3808	0.030*	
C37	0.7044 (2)	0.2096 (2)	0.38307 (11)	0.0205 (5)	
H37A	0.6940	0.2101	0.3410	0.025*	
C38	0.4412 (2)	0.5069 (2)	0.36453 (10)	0.0156 (4)	
C39	0.5592 (2)	0.5330 (2)	0.37148 (11)	0.0206 (5)	
H39A	0.6341	0.4731	0.3894	0.025*	
C40	0.5655 (3)	0.6487 (2)	0.35168 (11)	0.0261 (5)	
H40A	0.6450	0.6651	0.3560	0.031*	
C41	0.4541 (3)	0.7389 (2)	0.32574 (11)	0.0277 (6)	
H41A	0.4584	0.8162	0.3131	0.033*	
C42	0.3359 (3)	0.7140 (2)	0.31854 (11)	0.0256 (5)	
H42A	0.2606	0.7746	0.3013	0.031*	
C43	0.3305 (3)	0.5985 (2)	0.33716 (11)	0.0204 (5)	
H43A	0.2519	0.5817	0.3314	0.024*	
C44	0.2190 (2)	0.0905 (2)	0.27097 (10)	0.0168 (4)	
C45	0.4604 (2)	0.0246 (2)	0.20180 (10)	0.0182 (5)	
C46	0.3947 (2)	0.2583 (2)	0.14876 (10)	0.0167 (4)	
C47	0.2710 (2)	0.4822 (2)	0.21067 (10)	0.0173 (4)	
C48	0.0229 (2)	0.5545 (2)	0.27574 (10)	0.0182 (5)	
C49	0.0088 (2)	0.3253 (2)	0.26981 (10)	0.0170 (4)	
C50	0.2029 (2)	0.2631 (2)	0.35747 (10)	0.0175 (4)	
C51	0.4774 (2)	0.1058 (2)	0.33704 (10)	0.0178 (4)	
C52	0.5059 (2)	0.3065 (2)	0.25589 (10)	0.0173 (4)	
C53	0.3141 (3)	0.0748 (3)	0.49814 (14)	0.0371 (7)	
H53A	0.3683	0.0021	0.4780	0.056*	
H53B	0.2189	0.0929	0.4918	0.056*	
H53C	0.3283	0.0614	0.5417	0.056*	

### Atomic displacement parameters (Å^2^)

**Table:**

	*U*^11^	*U*^22^	*U*^33^	*U*^12^	*U*^13^	*U*^23^
Ru1	0.01299 (8)	0.01047 (8)	0.01293 (8)	−0.00370 (6)	−0.00310 (6)	−0.00082 (6)
Ru2	0.01320 (8)	0.01058 (8)	0.01226 (8)	−0.00360 (6)	−0.00261 (6)	−0.00003 (6)
Ru3	0.01372 (8)	0.01118 (8)	0.01253 (8)	−0.00423 (6)	−0.00349 (6)	−0.00038 (6)
As1	0.01311 (10)	0.01034 (10)	0.01269 (10)	−0.00425 (8)	−0.00292 (8)	−0.00022 (8)
As2	0.01245 (10)	0.01009 (10)	0.01241 (10)	−0.00383 (8)	−0.00205 (8)	0.00073 (8)
P1	0.0157 (3)	0.0133 (3)	0.0136 (3)	−0.0060 (2)	−0.0029 (2)	−0.0005 (2)
O1	0.0313 (10)	0.0192 (8)	0.0195 (8)	−0.0113 (7)	−0.0010 (7)	0.0004 (7)
O2	0.0214 (9)	0.0183 (9)	0.0372 (10)	0.0009 (7)	−0.0057 (8)	−0.0051 (8)
O3	0.0232 (9)	0.0246 (9)	0.0219 (9)	−0.0087 (7)	0.0018 (7)	−0.0004 (7)
O4	0.0202 (9)	0.0234 (9)	0.0297 (9)	−0.0109 (7)	−0.0069 (7)	0.0044 (7)
O5	0.0387 (11)	0.0162 (9)	0.0269 (10)	−0.0024 (8)	0.0089 (8)	−0.0027 (7)
O6	0.0216 (9)	0.0260 (9)	0.0261 (9)	−0.0115 (7)	−0.0050 (7)	0.0080 (7)
O7	0.0203 (9)	0.0317 (9)	0.0161 (8)	−0.0104 (7)	−0.0009 (7)	−0.0025 (7)
O8	0.0355 (11)	0.0154 (8)	0.0341 (10)	−0.0043 (8)	−0.0139 (8)	0.0024 (8)
O9	0.0191 (8)	0.0248 (9)	0.0234 (9)	−0.0079 (7)	−0.0009 (7)	0.0033 (7)
O10	0.0321 (10)	0.0238 (9)	0.0223 (9)	−0.0165 (8)	−0.0063 (7)	0.0067 (7)
C1	0.0175 (10)	0.0102 (9)	0.0135 (10)	−0.0064 (8)	0.0001 (8)	0.0000 (8)
C2	0.0170 (11)	0.0157 (10)	0.0170 (11)	−0.0062 (9)	−0.0036 (8)	0.0009 (9)
C3	0.0165 (11)	0.0152 (10)	0.0212 (11)	−0.0036 (9)	0.0000 (9)	−0.0024 (9)
C4	0.0246 (12)	0.0168 (11)	0.0158 (11)	−0.0103 (9)	0.0003 (9)	−0.0024 (9)
C5	0.0209 (11)	0.0181 (11)	0.0159 (11)	−0.0074 (9)	−0.0067 (9)	0.0016 (9)
C6	0.0157 (11)	0.0137 (10)	0.0202 (11)	−0.0044 (8)	−0.0038 (9)	0.0002 (9)
C7	0.0201 (11)	0.0152 (10)	0.0121 (10)	−0.0090 (9)	−0.0014 (8)	−0.0016 (8)
C8	0.0181 (11)	0.0162 (11)	0.0193 (11)	−0.0069 (9)	−0.0039 (9)	−0.0005 (9)
C9	0.0282 (13)	0.0149 (11)	0.0190 (11)	−0.0096 (10)	−0.0054 (9)	0.0020 (9)
C10	0.0267 (13)	0.0216 (12)	0.0232 (12)	−0.0146 (10)	0.0000 (10)	0.0010 (10)
C11	0.0195 (12)	0.0229 (12)	0.0256 (12)	−0.0090 (10)	0.0031 (10)	−0.0020 (10)
C12	0.0181 (11)	0.0147 (10)	0.0211 (11)	−0.0045 (9)	−0.0020 (9)	−0.0008 (9)
C13	0.0153 (10)	0.0115 (10)	0.0163 (10)	−0.0036 (8)	−0.0047 (8)	0.0001 (8)
C14	0.0141 (10)	0.0132 (10)	0.0150 (10)	−0.0036 (8)	−0.0034 (8)	−0.0011 (8)
C15	0.0172 (11)	0.0186 (11)	0.0207 (11)	−0.0059 (9)	−0.0034 (9)	0.0037 (9)
C16	0.0191 (11)	0.0235 (12)	0.0234 (12)	−0.0103 (10)	0.0009 (9)	−0.0035 (10)
C17	0.0133 (11)	0.0198 (11)	0.0317 (13)	−0.0020 (9)	−0.0035 (10)	−0.0049 (10)
C18	0.0234 (12)	0.0156 (11)	0.0255 (12)	−0.0034 (9)	−0.0065 (10)	0.0004 (10)
C19	0.0182 (11)	0.0134 (10)	0.0203 (11)	−0.0060 (9)	−0.0024 (9)	0.0012 (9)
C20	0.0139 (10)	0.0157 (10)	0.0146 (10)	−0.0068 (8)	−0.0032 (8)	0.0034 (8)
C21	0.0170 (11)	0.0167 (10)	0.0162 (10)	−0.0064 (9)	−0.0020 (8)	0.0002 (9)
C22	0.0222 (12)	0.0167 (11)	0.0243 (12)	−0.0094 (9)	−0.0043 (10)	0.0031 (9)
C23	0.0200 (12)	0.0275 (13)	0.0219 (12)	−0.0110 (10)	−0.0015 (9)	0.0070 (10)
C24	0.0201 (12)	0.0288 (13)	0.0164 (11)	−0.0065 (10)	0.0022 (9)	−0.0010 (10)
C25	0.0196 (11)	0.0173 (11)	0.0179 (11)	−0.0073 (9)	−0.0013 (9)	−0.0017 (9)
C26	0.0173 (11)	0.0231 (11)	0.0135 (10)	−0.0096 (9)	−0.0032 (8)	−0.0018 (9)
C27	0.0192 (12)	0.0291 (13)	0.0200 (12)	−0.0080 (10)	−0.0036 (9)	−0.0050 (10)
C28	0.0237 (13)	0.0435 (16)	0.0221 (13)	−0.0072 (12)	−0.0014 (10)	−0.0108 (12)
C29	0.0232 (14)	0.062 (2)	0.0147 (12)	−0.0135 (14)	0.0026 (10)	−0.0069 (13)
C30	0.0311 (14)	0.0494 (17)	0.0165 (12)	−0.0219 (13)	−0.0034 (10)	0.0071 (12)
C31	0.0243 (12)	0.0323 (13)	0.0163 (11)	−0.0162 (11)	−0.0074 (9)	0.0024 (10)
C32	0.0185 (11)	0.0134 (10)	0.0200 (11)	−0.0082 (9)	−0.0072 (9)	0.0033 (9)
C33	0.0269 (13)	0.0172 (11)	0.0208 (12)	−0.0069 (10)	−0.0077 (10)	−0.0007 (9)
C34	0.0332 (14)	0.0222 (12)	0.0253 (13)	−0.0102 (11)	−0.0140 (11)	0.0022 (10)
C35	0.0241 (13)	0.0208 (12)	0.0338 (14)	−0.0077 (10)	−0.0142 (11)	0.0090 (11)
C36	0.0176 (12)	0.0218 (12)	0.0341 (14)	−0.0061 (10)	−0.0024 (10)	0.0043 (11)
C37	0.0201 (12)	0.0241 (12)	0.0189 (11)	−0.0102 (10)	−0.0035 (9)	0.0035 (10)
C38	0.0222 (11)	0.0146 (10)	0.0121 (10)	−0.0083 (9)	−0.0019 (8)	−0.0028 (8)
C39	0.0228 (12)	0.0210 (12)	0.0206 (11)	−0.0106 (10)	−0.0049 (9)	0.0015 (9)
C40	0.0311 (14)	0.0282 (13)	0.0261 (13)	−0.0191 (11)	−0.0056 (11)	0.0014 (11)
C41	0.0477 (17)	0.0179 (12)	0.0223 (12)	−0.0176 (12)	−0.0048 (11)	0.0022 (10)
C42	0.0351 (15)	0.0170 (11)	0.0233 (12)	−0.0059 (11)	−0.0108 (11)	0.0012 (10)
C43	0.0249 (12)	0.0164 (11)	0.0209 (12)	−0.0073 (9)	−0.0064 (9)	−0.0015 (9)
C44	0.0199 (11)	0.0132 (10)	0.0165 (11)	−0.0039 (9)	−0.0042 (9)	−0.0022 (9)
C45	0.0213 (12)	0.0183 (11)	0.0182 (11)	−0.0098 (9)	−0.0027 (9)	−0.0037 (9)
C46	0.0175 (11)	0.0140 (10)	0.0171 (11)	−0.0028 (9)	−0.0029 (9)	−0.0033 (9)
C47	0.0161 (11)	0.0170 (11)	0.0182 (11)	−0.0038 (9)	−0.0043 (9)	−0.0031 (9)
C48	0.0221 (12)	0.0171 (11)	0.0161 (11)	−0.0088 (9)	0.0003 (9)	0.0021 (9)
C49	0.0193 (11)	0.0155 (10)	0.0138 (10)	−0.0026 (9)	−0.0064 (9)	0.0022 (9)
C50	0.0188 (11)	0.0169 (11)	0.0169 (11)	−0.0044 (9)	−0.0060 (9)	−0.0044 (9)
C51	0.0221 (12)	0.0178 (11)	0.0158 (11)	−0.0089 (9)	−0.0049 (9)	−0.0011 (9)
C52	0.0209 (11)	0.0141 (10)	0.0159 (11)	−0.0041 (9)	−0.0058 (9)	0.0002 (9)
C53	0.0394 (16)	0.0335 (15)	0.0441 (17)	−0.0230 (14)	−0.0149 (13)	0.0211 (13)

### Geometric parameters (Å, °)

**Table:**

Ru1—C45	1.890 (2)	C13—H13B	0.9700
Ru1—C46	1.925 (2)	C14—C19	1.392 (3)
Ru1—C44	1.941 (2)	C14—C15	1.395 (3)
Ru1—As1	2.4423 (3)	C15—C16	1.384 (3)
Ru1—Ru3	2.8471 (2)	C15—H15A	0.9300
Ru1—Ru2	2.8616 (2)	C16—C17	1.385 (3)
Ru2—C48	1.887 (2)	C16—H16A	0.9300
Ru2—C47	1.933 (2)	C17—C18	1.386 (3)
Ru2—C49	1.934 (2)	C17—H17A	0.9300
Ru2—As2	2.4209 (3)	C18—C19	1.389 (3)
Ru2—Ru3	2.8830 (2)	C18—H18A	0.9300
Ru3—C51	1.885 (2)	C19—H19A	0.9300
Ru3—C50	1.933 (2)	C20—C25	1.386 (3)
Ru3—C52	1.939 (2)	C20—C21	1.397 (3)
Ru3—P1	2.3495 (6)	C21—C22	1.391 (3)
As1—C7	1.952 (2)	C21—H21A	0.9300
As1—C1	1.953 (2)	C22—C23	1.380 (3)
As1—C13	1.968 (2)	C22—H22A	0.9300
As2—C14	1.939 (2)	C23—C24	1.385 (3)
As2—C20	1.940 (2)	C23—H23A	0.9300
As2—C13	1.965 (2)	C24—C25	1.396 (3)
P1—C26	1.822 (2)	C24—H24A	0.9300
P1—C38	1.833 (2)	C25—H25A	0.9300
P1—C32	1.838 (2)	C26—C27	1.396 (3)
O1—C44	1.139 (3)	C26—C31	1.407 (3)
O2—C45	1.148 (3)	C27—C28	1.389 (3)
O3—C46	1.150 (3)	C27—H27A	0.9300
O4—C47	1.141 (3)	C28—C29	1.375 (4)
O5—C48	1.145 (3)	C28—H28A	0.9300
O6—C49	1.146 (3)	C29—C30	1.388 (4)
O7—C50	1.148 (3)	C29—H29A	0.9300
O8—C51	1.144 (3)	C30—C31	1.389 (4)
O9—C52	1.142 (3)	C30—H30A	0.9300
O10—C31	1.368 (3)	C32—C37	1.395 (3)
O10—C53	1.436 (3)	C32—C33	1.395 (3)
C1—C2	1.391 (3)	C33—C34	1.392 (3)
C1—C6	1.399 (3)	C33—H33A	0.9300
C2—C3	1.389 (3)	C34—C35	1.381 (4)
C2—H2A	0.9300	C34—H34A	0.9300
C3—C4	1.387 (3)	C35—C36	1.378 (4)
C3—H3A	0.9300	C35—H35A	0.9300
C4—C5	1.383 (3)	C36—C37	1.394 (3)
C4—H4A	0.9300	C36—H36A	0.9300
C5—C6	1.383 (3)	C37—H37A	0.9300
C5—H5A	0.9300	C38—C39	1.393 (3)
C6—H6A	0.9300	C38—C43	1.398 (3)
C7—C12	1.386 (3)	C39—C40	1.395 (3)
C7—C8	1.401 (3)	C39—H39A	0.9300
C8—C9	1.387 (3)	C40—C41	1.382 (4)
C8—H8A	0.9300	C40—H40A	0.9300
C9—C10	1.391 (3)	C41—C42	1.389 (4)
C9—H9A	0.9300	C41—H41A	0.9300
C10—C11	1.389 (3)	C42—C43	1.385 (3)
C10—H10A	0.9300	C42—H42A	0.9300
C11—C12	1.395 (3)	C43—H43A	0.9300
C11—H11A	0.9300	C53—H53A	0.9600
C12—H12A	0.9300	C53—H53B	0.9600
C13—H13A	0.9700	C53—H53C	0.9600
			
C45—Ru1—C46	91.04 (9)	C19—C14—C15	119.3 (2)
C45—Ru1—C44	90.46 (9)	C19—C14—As2	123.36 (16)
C46—Ru1—C44	172.53 (9)	C15—C14—As2	117.35 (16)
C45—Ru1—As1	107.84 (7)	C16—C15—C14	120.7 (2)
C46—Ru1—As1	95.19 (6)	C16—C15—H15A	119.6
C44—Ru1—As1	91.31 (6)	C14—C15—H15A	119.6
C45—Ru1—Ru3	98.86 (7)	C15—C16—C17	119.7 (2)
C46—Ru1—Ru3	91.92 (6)	C15—C16—H16A	120.2
C44—Ru1—Ru3	80.62 (6)	C17—C16—H16A	120.2
As1—Ru1—Ru3	152.186 (9)	C16—C17—C18	120.1 (2)
C45—Ru1—Ru2	158.58 (6)	C16—C17—H17A	120.0
C46—Ru1—Ru2	83.95 (7)	C18—C17—H17A	120.0
C44—Ru1—Ru2	92.01 (7)	C17—C18—C19	120.4 (2)
As1—Ru1—Ru2	93.368 (8)	C17—C18—H18A	119.8
Ru3—Ru1—Ru2	60.666 (6)	C19—C18—H18A	119.8
C48—Ru2—C47	91.34 (9)	C18—C19—C14	119.8 (2)
C48—Ru2—C49	91.77 (9)	C18—C19—H19A	120.1
C47—Ru2—C49	175.54 (9)	C14—C19—H19A	120.1
C48—Ru2—As2	97.50 (7)	C25—C20—C21	119.46 (19)
C47—Ru2—As2	91.55 (6)	C25—C20—As2	122.17 (16)
C49—Ru2—As2	91.22 (6)	C21—C20—As2	117.89 (16)
C48—Ru2—Ru1	169.88 (7)	C22—C21—C20	120.1 (2)
C47—Ru2—Ru1	93.48 (7)	C22—C21—H21A	120.0
C49—Ru2—Ru1	82.96 (7)	C20—C21—H21A	120.0
As2—Ru2—Ru1	91.262 (8)	C23—C22—C21	120.2 (2)
C48—Ru2—Ru3	113.31 (7)	C23—C22—H22A	119.9
C47—Ru2—Ru3	76.67 (6)	C21—C22—H22A	119.9
C49—Ru2—Ru3	99.15 (6)	C22—C23—C24	120.2 (2)
As2—Ru2—Ru3	146.953 (10)	C22—C23—H23A	119.9
Ru1—Ru2—Ru3	59.420 (6)	C24—C23—H23A	119.9
C51—Ru3—C50	95.57 (10)	C23—C24—C25	119.9 (2)
C51—Ru3—C52	97.26 (10)	C23—C24—H24A	120.1
C50—Ru3—C52	167.15 (9)	C25—C24—H24A	120.1
C51—Ru3—P1	93.36 (7)	C20—C25—C24	120.2 (2)
C50—Ru3—P1	90.24 (6)	C20—C25—H25A	119.9
C52—Ru3—P1	88.34 (7)	C24—C25—H25A	119.9
C51—Ru3—Ru1	89.47 (7)	C27—C26—C31	118.2 (2)
C50—Ru3—Ru1	93.66 (6)	C27—C26—P1	122.46 (17)
C52—Ru3—Ru1	87.15 (6)	C31—C26—P1	119.37 (18)
P1—Ru3—Ru1	174.946 (15)	C28—C27—C26	121.4 (2)
C51—Ru3—Ru2	145.12 (6)	C28—C27—H27A	119.3
C50—Ru3—Ru2	72.13 (6)	C26—C27—H27A	119.3
C52—Ru3—Ru2	97.40 (6)	C29—C28—C27	119.2 (3)
P1—Ru3—Ru2	118.530 (16)	C29—C28—H28A	120.4
Ru1—Ru3—Ru2	59.915 (6)	C27—C28—H28A	120.4
C7—As1—C1	97.55 (9)	C28—C29—C30	121.2 (2)
C7—As1—C13	104.20 (9)	C28—C29—H29A	119.4
C1—As1—C13	100.98 (9)	C30—C29—H29A	119.4
C7—As1—Ru1	112.20 (6)	C29—C30—C31	119.5 (2)
C1—As1—Ru1	122.94 (6)	C29—C30—H30A	120.3
C13—As1—Ru1	116.04 (6)	C31—C30—H30A	120.3
C14—As2—C20	104.26 (9)	O10—C31—C30	124.8 (2)
C14—As2—C13	99.21 (9)	O10—C31—C26	114.7 (2)
C20—As2—C13	104.44 (9)	C30—C31—C26	120.5 (2)
C14—As2—Ru2	116.46 (6)	C37—C32—C33	118.9 (2)
C20—As2—Ru2	114.03 (6)	C37—C32—P1	120.28 (17)
C13—As2—Ru2	116.51 (6)	C33—C32—P1	120.79 (18)
C26—P1—C38	103.46 (10)	C34—C33—C32	120.1 (2)
C26—P1—C32	102.75 (10)	C34—C33—H33A	120.0
C38—P1—C32	102.83 (10)	C32—C33—H33A	120.0
C26—P1—Ru3	117.33 (7)	C35—C34—C33	120.4 (2)
C38—P1—Ru3	113.44 (7)	C35—C34—H34A	119.8
C32—P1—Ru3	115.22 (7)	C33—C34—H34A	119.8
C31—O10—C53	117.7 (2)	C36—C35—C34	120.1 (2)
C2—C1—C6	119.1 (2)	C36—C35—H35A	119.9
C2—C1—As1	120.56 (16)	C34—C35—H35A	119.9
C6—C1—As1	120.17 (16)	C35—C36—C37	119.9 (2)
C3—C2—C1	120.2 (2)	C35—C36—H36A	120.0
C3—C2—H2A	119.9	C37—C36—H36A	120.0
C1—C2—H2A	119.9	C36—C37—C32	120.6 (2)
C4—C3—C2	120.0 (2)	C36—C37—H37A	119.7
C4—C3—H3A	120.0	C32—C37—H37A	119.7
C2—C3—H3A	120.0	C39—C38—C43	118.6 (2)
C5—C4—C3	120.2 (2)	C39—C38—P1	122.91 (18)
C5—C4—H4A	119.9	C43—C38—P1	118.50 (16)
C3—C4—H4A	119.9	C38—C39—C40	120.3 (2)
C6—C5—C4	119.8 (2)	C38—C39—H39A	119.9
C6—C5—H5A	120.1	C40—C39—H39A	119.9
C4—C5—H5A	120.1	C41—C40—C39	120.4 (2)
C5—C6—C1	120.6 (2)	C41—C40—H40A	119.8
C5—C6—H6A	119.7	C39—C40—H40A	119.8
C1—C6—H6A	119.7	C40—C41—C42	119.9 (2)
C12—C7—C8	119.4 (2)	C40—C41—H41A	120.0
C12—C7—As1	125.02 (16)	C42—C41—H41A	120.0
C8—C7—As1	115.46 (16)	C43—C42—C41	119.8 (2)
C9—C8—C7	120.5 (2)	C43—C42—H42A	120.1
C9—C8—H8A	119.7	C41—C42—H42A	120.1
C7—C8—H8A	119.7	C42—C43—C38	121.1 (2)
C8—C9—C10	120.0 (2)	C42—C43—H43A	119.5
C8—C9—H9A	120.0	C38—C43—H43A	119.5
C10—C9—H9A	120.0	O1—C44—Ru1	174.8 (2)
C11—C10—C9	119.5 (2)	O2—C45—Ru1	175.9 (2)
C11—C10—H10A	120.2	O3—C46—Ru1	174.4 (2)
C9—C10—H10A	120.2	O4—C47—Ru2	170.1 (2)
C10—C11—C12	120.6 (2)	O5—C48—Ru2	176.4 (2)
C10—C11—H11A	119.7	O6—C49—Ru2	173.66 (19)
C12—C11—H11A	119.7	O7—C50—Ru3	171.28 (19)
C7—C12—C11	119.9 (2)	O8—C51—Ru3	176.9 (2)
C7—C12—H12A	120.0	O9—C52—Ru3	174.72 (19)
C11—C12—H12A	120.0	O10—C53—H53A	109.5
As2—C13—As1	111.74 (10)	O10—C53—H53B	109.5
As2—C13—H13A	109.3	H53A—C53—H53B	109.5
As1—C13—H13A	109.3	O10—C53—H53C	109.5
As2—C13—H13B	109.3	H53A—C53—H53C	109.5
As1—C13—H13B	109.3	H53B—C53—H53C	109.5
H13A—C13—H13B	107.9		
			
C45—Ru1—Ru2—C48	−64.4 (4)	C7—As1—C1—C2	−106.30 (17)
C46—Ru1—Ru2—C48	−141.6 (4)	C13—As1—C1—C2	147.58 (16)
C44—Ru1—Ru2—C48	32.1 (4)	Ru1—As1—C1—C2	16.38 (19)
As1—Ru1—Ru2—C48	123.5 (4)	C7—As1—C1—C6	68.64 (18)
Ru3—Ru1—Ru2—C48	−46.0 (4)	C13—As1—C1—C6	−37.49 (18)
C45—Ru1—Ru2—C47	53.9 (2)	Ru1—As1—C1—C6	−168.68 (13)
C46—Ru1—Ru2—C47	−23.35 (9)	C6—C1—C2—C3	0.3 (3)
C44—Ru1—Ru2—C47	150.35 (9)	As1—C1—C2—C3	175.31 (16)
As1—Ru1—Ru2—C47	−118.22 (6)	C1—C2—C3—C4	1.1 (3)
Ru3—Ru1—Ru2—C47	72.29 (6)	C2—C3—C4—C5	−1.0 (3)
C45—Ru1—Ru2—C49	−123.4 (2)	C3—C4—C5—C6	−0.4 (3)
C46—Ru1—Ru2—C49	159.35 (9)	C4—C5—C6—C1	1.8 (3)
C44—Ru1—Ru2—C49	−26.95 (9)	C2—C1—C6—C5	−1.8 (3)
As1—Ru1—Ru2—C49	64.48 (6)	As1—C1—C6—C5	−176.76 (16)
Ru3—Ru1—Ru2—C49	−105.01 (6)	C1—As1—C7—C12	−120.94 (19)
C45—Ru1—Ru2—As2	145.56 (19)	C13—As1—C7—C12	−17.5 (2)
C46—Ru1—Ru2—As2	68.28 (6)	Ru1—As1—C7—C12	108.78 (18)
C44—Ru1—Ru2—As2	−118.02 (6)	C1—As1—C7—C8	63.38 (18)
As1—Ru1—Ru2—As2	−26.588 (9)	C13—As1—C7—C8	166.78 (16)
Ru3—Ru1—Ru2—As2	163.916 (9)	Ru1—As1—C7—C8	−66.90 (17)
C45—Ru1—Ru2—Ru3	−18.36 (19)	C12—C7—C8—C9	0.5 (3)
C46—Ru1—Ru2—Ru3	−95.63 (6)	As1—C7—C8—C9	176.41 (17)
C44—Ru1—Ru2—Ru3	78.06 (6)	C7—C8—C9—C10	0.3 (3)
As1—Ru1—Ru2—Ru3	169.496 (9)	C8—C9—C10—C11	−0.6 (4)
C45—Ru1—Ru3—C51	−24.23 (10)	C9—C10—C11—C12	0.1 (4)
C46—Ru1—Ru3—C51	−115.58 (10)	C8—C7—C12—C11	−0.9 (3)
C44—Ru1—Ru3—C51	64.77 (10)	As1—C7—C12—C11	−176.44 (17)
As1—Ru1—Ru3—C51	139.50 (7)	C10—C11—C12—C7	0.6 (4)
Ru2—Ru1—Ru3—C51	162.45 (7)	C14—As2—C13—As1	−148.46 (11)
C45—Ru1—Ru3—C50	−119.78 (9)	C20—As2—C13—As1	104.11 (11)
C46—Ru1—Ru3—C50	148.86 (9)	Ru2—As2—C13—As1	−22.64 (13)
C44—Ru1—Ru3—C50	−30.78 (9)	C7—As1—C13—As2	120.32 (11)
As1—Ru1—Ru3—C50	43.95 (7)	C1—As1—C13—As2	−138.89 (10)
Ru2—Ru1—Ru3—C50	66.90 (7)	Ru1—As1—C13—As2	−3.55 (13)
C45—Ru1—Ru3—C52	73.07 (9)	C20—As2—C14—C19	0.7 (2)
C46—Ru1—Ru3—C52	−18.29 (9)	C13—As2—C14—C19	−106.86 (19)
C44—Ru1—Ru3—C52	162.07 (9)	Ru2—As2—C14—C19	127.29 (17)
As1—Ru1—Ru3—C52	−123.20 (7)	C20—As2—C14—C15	179.39 (17)
Ru2—Ru1—Ru3—C52	−100.25 (7)	C13—As2—C14—C15	71.82 (18)
C45—Ru1—Ru3—Ru2	173.32 (7)	Ru2—As2—C14—C15	−54.04 (18)
C46—Ru1—Ru3—Ru2	81.96 (7)	C19—C14—C15—C16	2.2 (3)
C44—Ru1—Ru3—Ru2	−97.68 (7)	As2—C14—C15—C16	−176.57 (18)
As1—Ru1—Ru3—Ru2	−22.956 (18)	C14—C15—C16—C17	−0.4 (3)
C48—Ru2—Ru3—C51	140.28 (14)	C15—C16—C17—C18	−1.7 (4)
C47—Ru2—Ru3—C51	−134.08 (14)	C16—C17—C18—C19	2.0 (4)
C49—Ru2—Ru3—C51	44.34 (14)	C17—C18—C19—C14	−0.2 (3)
As2—Ru2—Ru3—C51	−62.34 (12)	C15—C14—C19—C18	−1.8 (3)
Ru1—Ru2—Ru3—C51	−31.81 (12)	As2—C14—C19—C18	176.81 (17)
C48—Ru2—Ru3—C50	66.77 (10)	C14—As2—C20—C25	−126.47 (18)
C47—Ru2—Ru3—C50	152.42 (10)	C13—As2—C20—C25	−22.8 (2)
C49—Ru2—Ru3—C50	−29.16 (10)	Ru2—As2—C20—C25	105.46 (17)
As2—Ru2—Ru3—C50	−135.84 (7)	C14—As2—C20—C21	61.52 (18)
Ru1—Ru2—Ru3—C50	−105.32 (7)	C13—As2—C20—C21	165.16 (16)
C48—Ru2—Ru3—C52	−105.57 (10)	Ru2—As2—C20—C21	−66.56 (18)
C47—Ru2—Ru3—C52	−19.92 (10)	C25—C20—C21—C22	0.7 (3)
C49—Ru2—Ru3—C52	158.50 (9)	As2—C20—C21—C22	172.95 (16)
As2—Ru2—Ru3—C52	51.82 (7)	C20—C21—C22—C23	−0.7 (3)
Ru1—Ru2—Ru3—C52	82.34 (7)	C21—C22—C23—C24	0.4 (3)
C48—Ru2—Ru3—P1	−13.43 (7)	C22—C23—C24—C25	−0.1 (4)
C47—Ru2—Ru3—P1	72.22 (7)	C21—C20—C25—C24	−0.4 (3)
C49—Ru2—Ru3—P1	−109.36 (7)	As2—C20—C25—C24	−172.32 (17)
As2—Ru2—Ru3—P1	143.96 (2)	C23—C24—C25—C20	0.1 (3)
Ru1—Ru2—Ru3—P1	174.484 (18)	C38—P1—C26—C27	6.5 (2)
C48—Ru2—Ru3—Ru1	172.09 (7)	C32—P1—C26—C27	113.28 (19)
C47—Ru2—Ru3—Ru1	−102.26 (7)	Ru3—P1—C26—C27	−119.22 (17)
C49—Ru2—Ru3—Ru1	76.15 (7)	C38—P1—C26—C31	−175.21 (17)
As2—Ru2—Ru3—Ru1	−30.525 (15)	C32—P1—C26—C31	−68.45 (19)
C45—Ru1—As1—C7	85.03 (10)	Ru3—P1—C26—C31	59.04 (19)
C46—Ru1—As1—C7	177.80 (10)	C31—C26—C27—C28	−1.8 (3)
C44—Ru1—As1—C7	−5.88 (10)	P1—C26—C27—C28	176.48 (18)
Ru3—Ru1—As1—C7	−78.06 (7)	C26—C27—C28—C29	−0.2 (4)
Ru2—Ru1—As1—C7	−97.98 (7)	C27—C28—C29—C30	1.4 (4)
C45—Ru1—As1—C1	−30.66 (10)	C28—C29—C30—C31	−0.6 (4)
C46—Ru1—As1—C1	62.12 (10)	C53—O10—C31—C30	11.7 (3)
C44—Ru1—As1—C1	−121.57 (10)	C53—O10—C31—C26	−169.1 (2)
Ru3—Ru1—As1—C1	166.25 (7)	C29—C30—C31—O10	177.8 (2)
Ru2—Ru1—As1—C1	146.34 (7)	C29—C30—C31—C26	−1.4 (4)
C45—Ru1—As1—C13	−155.35 (10)	C27—C26—C31—O10	−176.68 (19)
C46—Ru1—As1—C13	−62.58 (10)	P1—C26—C31—O10	5.0 (3)
C44—Ru1—As1—C13	113.73 (10)	C27—C26—C31—C30	2.6 (3)
Ru3—Ru1—As1—C13	41.55 (8)	P1—C26—C31—C30	−175.73 (18)
Ru2—Ru1—As1—C13	21.64 (7)	C26—P1—C32—C37	168.47 (18)
C48—Ru2—As2—C14	−26.10 (10)	C38—P1—C32—C37	−84.30 (19)
C47—Ru2—As2—C14	−117.66 (10)	Ru3—P1—C32—C37	39.64 (19)
C49—Ru2—As2—C14	65.84 (10)	C26—P1—C32—C33	−14.2 (2)
Ru1—Ru2—As2—C14	148.83 (7)	C38—P1—C32—C33	93.00 (19)
Ru3—Ru2—As2—C14	174.76 (7)	Ru3—P1—C32—C33	−143.06 (16)
C48—Ru2—As2—C20	95.45 (10)	C37—C32—C33—C34	−0.3 (3)
C47—Ru2—As2—C20	3.89 (10)	P1—C32—C33—C34	−177.61 (17)
C49—Ru2—As2—C20	−172.61 (10)	C32—C33—C34—C35	1.4 (4)
Ru1—Ru2—As2—C20	−89.62 (7)	C33—C34—C35—C36	−1.4 (4)
Ru3—Ru2—As2—C20	−63.69 (8)	C34—C35—C36—C37	0.3 (4)
C48—Ru2—As2—C13	−142.72 (10)	C35—C36—C37—C32	0.8 (3)
C47—Ru2—As2—C13	125.73 (10)	C33—C32—C37—C36	−0.8 (3)
C49—Ru2—As2—C13	−50.77 (10)	P1—C32—C37—C36	176.54 (17)
Ru1—Ru2—As2—C13	32.21 (7)	C26—P1—C38—C39	107.4 (2)
Ru3—Ru2—As2—C13	58.15 (7)	C32—P1—C38—C39	0.7 (2)
C51—Ru3—P1—C26	−95.89 (11)	Ru3—P1—C38—C39	−124.40 (18)
C50—Ru3—P1—C26	−0.29 (11)	C26—P1—C38—C43	−73.14 (19)
C52—Ru3—P1—C26	166.93 (11)	C32—P1—C38—C43	−179.84 (18)
Ru2—Ru3—P1—C26	69.41 (9)	Ru3—P1—C38—C43	55.06 (19)
C51—Ru3—P1—C38	143.46 (11)	C43—C38—C39—C40	0.3 (3)
C50—Ru3—P1—C38	−120.94 (11)	P1—C38—C39—C40	179.77 (19)
C52—Ru3—P1—C38	46.28 (10)	C38—C39—C40—C41	0.9 (4)
Ru2—Ru3—P1—C38	−51.24 (8)	C39—C40—C41—C42	−0.9 (4)
C51—Ru3—P1—C32	25.31 (11)	C40—C41—C42—C43	−0.3 (4)
C50—Ru3—P1—C32	120.91 (10)	C41—C42—C43—C38	1.5 (4)
C52—Ru3—P1—C32	−71.87 (10)	C39—C38—C43—C42	−1.5 (3)
Ru2—Ru3—P1—C32	−169.39 (8)	P1—C38—C43—C42	179.03 (19)

### Hydrogen-bond geometry (Å, °)

**Table:**

*D*—H···*A*	*D*—H	H···*A*	*D*···*A*	*D*—H···*A*
C16—H16A···O4^i^	0.93	2.52	3.244 (3)	135
C29—H29A···O5^ii^	0.93	2.56	3.286 (3)	135
C5—H5A···Cg1^iii^	0.93	2.84	3.668 (2)	149
C18—H18A···Cg2^iv^	0.93	2.95	3.701 (2)	139
C22—H22A···Cg2^v^	0.93	2.95	3.787 (2)	151
C24—H24A···Cg3^iv^	0.93	2.81	3.499 (2)	132

Symmetry codes: (i) *x*−1, *y*, *z*; (ii) −*x*, −*y*+1, −*z*+1; (iii) −*x*, −*y*, −*z*; (iv) −*x*, −*y*+1, −*z*; (v) *x*, *y*+1, *z*.
